# Serotypes and Antibiotic Susceptibility of *Streptococcus pneumoniae* Isolates from Invasive Pneumococcal Disease and Asymptomatic Carriage in a Pre-vaccination Period, in Algeria

**DOI:** 10.3389/fmicb.2016.00803

**Published:** 2016-06-14

**Authors:** Hanifa Ziane, Vera Manageiro, Eugénia Ferreira, Inês B. Moura, Soumia Bektache, Mohamed Tazir, Manuela Caniça

**Affiliations:** ^1^Service de Microbiologie Médicale, Centre Hospitalo-Universitaire Mustapha Bacha, Faculté de MédecineAlgiers, Algeria; ^2^National Reference Laboratory of Antibiotic Resistances and Healthcare Associated Infections, Department of Infectious Diseases, National Institute of Health Doutor Ricardo JorgeLisbon, Portugal; ^3^Centre for the Studies of Animal Science, Institute of Agrarian and Agri-Food Sciences and Technologies, University of OportoOporto, Portugal

**Keywords:** pneumococci serotype, antibiotic susceptibility, invasive disease, pneumococcal carriage, children, vaccine, Algeria

## Abstract

In Algeria, few data is available concerning the distribution of pneumococcal serotypes and respective antibiotic resistance for the current pre-vaccination period, which is a public health concern. We identified the most frequent *Streptococcus pneumoniae* serogroup/types implicated in invasive pneumococcal disease (IPD; *n* = 80) and carriage (*n* = 138) in Algerian children younger than 5 years old. Serogroup/types of 78 IPD isolates were identified by capsular typing using a sequential multiplex PCR. Overall, serotypes 14, 19F, 6B, 23F, 18C, 1, 5, 7F, 19A, and 3 (55% of PCV7 serotypes, 71.3% of PCV10, and 90% of PCV13) were identified. Additionally, 7.5% of the non-vaccine serotypes 6C, 9N/L, 20, 24F, 35B, and 35F, were observed. In the case of *S. pneumoniae* asymptomatic children carriers, the most common serogroup/types were 6B, 14, 19F, 23F, 4, 9V/A, 1, 19A, 6A, and 3 (42.7% of PCV7 serotypes, 44.2% of PCV10, and 58% of PCV13). For 6.1% of the cases co-colonization was detected. Serotypes 14, 1, 5, and 19A were more implicated in IPD (*p* < 0.01), whereas serotype 6A was exclusively isolated from carriers (*p* < 0.01). Deaths associated with IPD were related to serotypes 19A, 14, 18C, and one non-typeable isolate. Among IPD related to vaccine serotypes, the rates of penicillin non-susceptible isolates were higher in no meningitis cases (80%) than in meningitis (66.7%), with serotypes 14, 19A, 19F, and 23F presenting the highest MIC levels (>2μg/ml). Resistance to cefotaxime was higher in isolates from meningitis (40.5%); however, resistance to erythromycin and co-trimoxazole (>40%) was more pronounced in no-meningeal forms. Overall, our results showed that PCV13 conjugate vaccine would cover up to 90% of the circulating isolates associated with IPD in Algeria, highlighting the importance of monitoring the frequency of *S. pneumoniae* serogroups/types during pre- and post-vaccination periods.

## Introduction

*Streptococcus pneumoniae* remains the leading cause of bacterial infection among children worldwide, being the most common cause of bacterial pneumonia, and an important cause of meningitis and bacteremia. Approximately 800,000 deaths per year occur among children as a result of pneumococcal infection (Johnson et al., 2010). The management of pneumococcal infections has been aggravated by the rapid worldwide increase of resistance to penicillin and other antibiotics, mostly related to the misuse of these drugs in respiratory pathogens (Song et al., 2012; Ginsburg et al., 2013).

Despite the diversity of capsular types, comprising at least 98 distinct serotypes, only some variants are associated with invasive pneumococcal disease (IPD; Caierão et al., 2014; Richter et al., 2014).

Pneumococcal disease is frequently preceded by asymptomatic nasopharyngeal colonization, which can be quite high in early childhood (Bogaert et al., 2004; Miernyk et al., 2011; Satzke et al., 2013). It is generally agreed that most serotypes recovered from IPD are also frequently identified in colonized healthy children (Bogaert et al., 2004; Dias and Caniça, 2007). However, serotype prevalence can change according to age, geography, time, and antibiotic resistance, among other factors (Finland and Barnes, 1977; Hausdorff et al., 2005; Ingels et al., 2012). IPD has also been related to recent respiratory viral infection (Weinberger et al., 2014).

In some countries, the use of effective pneumococcal conjugate vaccines (PCVs) during infancy have contributed to reduce morbidity and mortality associated to IPD, as well as nasopharyngeal colonization by vaccine serotypes (Ghaffar et al., 2004; Millar et al., 2006; Tan, 2012). Despite the availability of these vaccines, pneumococcal infections remain a global problem due to the replacement of vaccine by non-vaccine serotypes, mostly associated with the emergence of multidrug resistant serotypes, such as the serotype 19A (Dias and Caniça, 2007; Dagan et al., 2009; Richter et al., 2014). Thus, due to the widespread phenomena of serotype replacement, PCVs have been substituted by higher valence pneumococcal vaccines (from PCV7 and PCV10 to PCV13) across the world, according with the recommendations of the Centers for Disease Control and Prevention (CDC, 2013).

The monitoring of antibiotic resistance trends and serotype distribution in the pre- and post-vaccination periods is essential to assess the dynamic change of epidemiology. This way, the impact of vaccines and antibiotic use control programmes should be evaluated across countries.

In this study, we analyzed the frequency of serotypes associated to IPD and *S. pneumoniae* asymptomatic carriage among children younger than 5 years old in Algeria, before the introduction of the pneumococcal vaccine, and correlated the isolates antibiotic susceptibility with vaccine serotypes .

## Materials and methods

### Bacterial isolates

An overall of 218 *S. pneumoniae* isolates recovered from children <5 years old were collected and studied regarding the serotype. Briefly, 80 IPD isolates [children <1 year old (*n* = 44), 1–2 years old (*n* = 20), and 3–5 years old (*n* = 16)] were collected between the 1st January 2010 and the 31st December 2014. The isolates were recovered from routine microbiological cultures at Laboratory of Clinical Microbiology, at CHU Mustapha Bacha (LCM/CHU), Algiers, Algeria, and at cooperation laboratories in the north (*n* = 50), west (*n* = 12), south (*n* = 2), and northeast (*n* = 16) regions of the country that sent samples and/or isolates to LCM/CHU. Isolates were included if they were obtained from consecutive blood, and cerebrospinal, pleural, peritoneal, ascitic, bone, and joint fluids from patients with symptoms compatible with IPD. Only one isolate per patient was considered .

In addition, all nasopharyngeal cultures (*n* = 130) recovered at LCM/CHU between 2011 and in 2012, from asymptomatic children <5 years old were also included for serotyping evaluation. Each age group of <1 year, 1–2 years, and 3–5 years included 91, 20, and 27 *S. pneumoniae* isolates, respectively. Only one isolate per child was considered, except for eight cultures corresponding to carriers co-colonized with two pneumococcal serogroups/types (in a total of 138 isolates). Authorization for carriage study was approved by “The Direction de la Santé et de la Population de la Wilaya d'Alger,” in Algeria.

**Figure 1:**
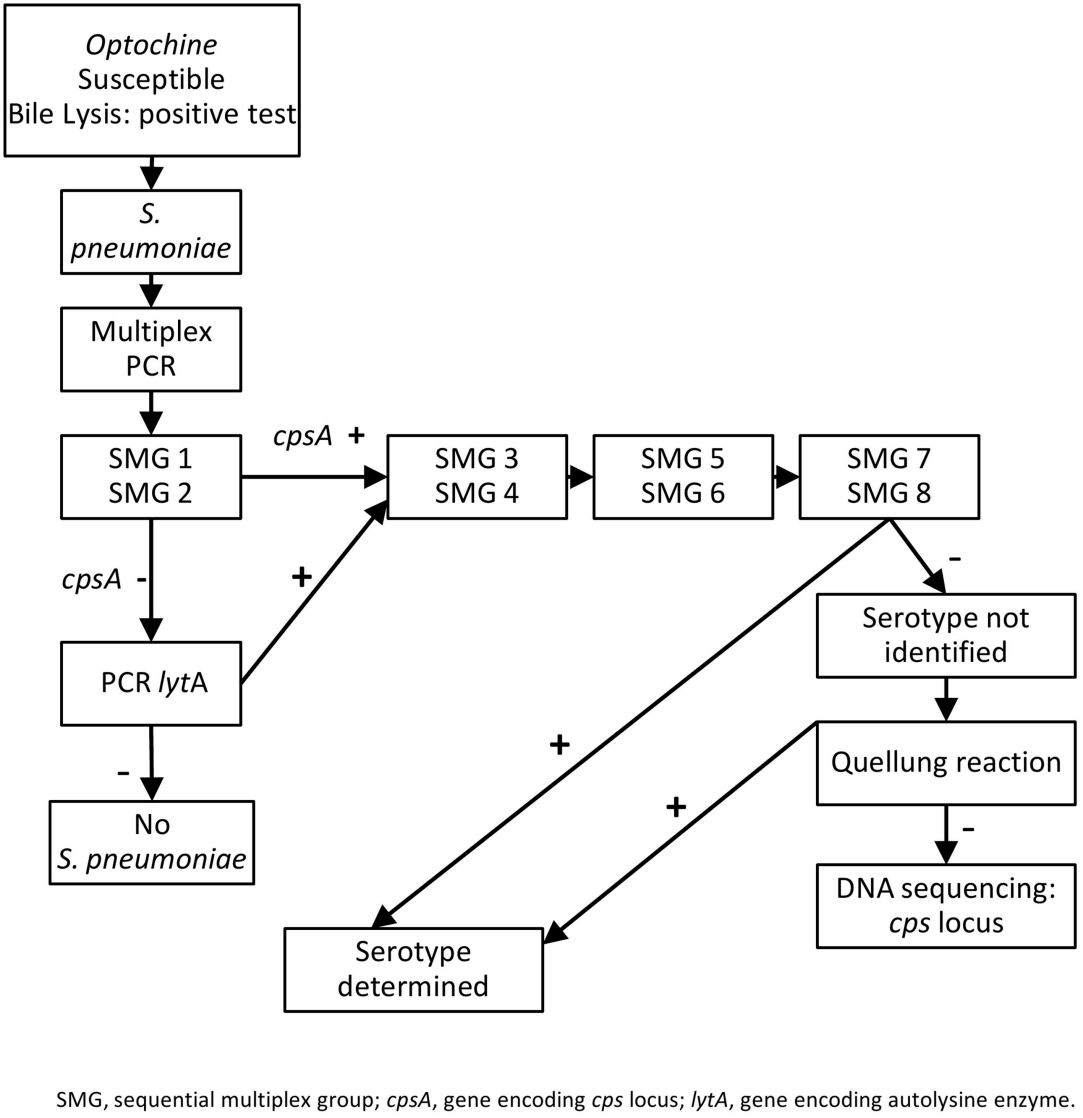
**Multiplex PCR scheme used in capsular typing of *S. pneumoniae* isolates from Algeria, and general scheme proposed for usual pneumococcal serotyping**.

Culture of sterile body fluids and nasopharyngeal samples at LCM/CHU was carried out by standard protocols. Pneumococcal isolates were identified using classic microbiological tests: colony morphology, optochin susceptibility, and bile solubility (Figure 1). Thirty seven *S*. *pneumoniae* isolates expressing different serogroups/types (1, 3, 4, 5, 6B, 6C, 7F, 7C, 8, 9V, 9N, 10A, 10F, 11A, 12F, 13, 14, 15A, 15B, 16F, 17F, 18C, 19F, 19A, 20, 21, 22F, 23A, 23B, 23F, 24F, 31, 33F, 34, 35A, 35B, and 35F), belonging to the collection of the National Reference Laboratory of Antibiotic Resistances and Healthcare Associated Infections, at National Institute of Health in Portugal, were also used as controls for serotype identification, as previously (Ziane et al., 2015).

### DNA extraction and multiplex PCRs

To extract DNA from clinical and control *S. pneumoniae* isolates, several colonies were picked from the culture plates. DNA was extracted by the heat lysis method and stored at −20°C until further analysis. The serogroups, and whenever possible the serotypes, of 80 *S. pneumoniae* from IPD and of 138 recovered in asymptomatic carriers were determined by sequential multiplex groups (SMGs), as previously described (Ziane et al., 2015; Figure 1).

### Quellung serotyping

In addition to multiplex PCR (Ziane et al., 2015), serogroups/types of all the 80 invasive isolates were determined at LCM/CHU by Quellung reaction with specific type antiserum [Statens-Serum Institute]. Concerning the isolates recovered from carriers, only those determined as serotype 6A/B or as non-typeable by the multiplex PCR method, were characterized by Quellung reaction (Figure 1).

### PCR detection of autolysin (*lytA*) gene

A primer pair 5′-TCCAGCCTGTAGCCATTTCG-3′ and 5′- GCGGTTGAACTGATTGAAAG-3′ that specifically targeted a 472 bp internal region of the autolysin (*lytA*) gene was used to the identification of *cps*-negative *S. pneumoniae*. The amplification conditions were: initial denaturation at 94°C for 5 min., followed by 30 cycles of denaturation at 94°C for 30 s, annealing at 56°C for 30 s and extension at 72°C for 30 s, and a final extension at 72°C for 5 min. Positive and negative controls were included in each PCR reaction.

### Antibiotic susceptibility testing

Susceptibility testing of 72 IPD vaccine serotype isolates and of 8 IPD non-vaccine serotype was carried out by an agar disk diffusion method for four antibiotics (erythromycin, clindamycin, co-trimoxazole, tetracycline), and minimal inhibitory concentration (MIC) was determined using *E*-test method (AB Biodisk) for four β-lactam antibiotics (penicillin, amoxicillin, cefotaxime, and imipenem). Testing conditions and susceptibility interpretation followed the standards proposed by the Clinical and Laboratory Standards Institute (CLSI, 2014). *S. pneumoniae* ATCC 49619 was used as the quality control strain.

### Statistical analysis

OpenEpi software, version 3.03a (Dean et al., 2015), was used for statistical analysis. Fisher exact test was used to assess differences between IPD and carriers groups. One-tail *P* ≤ 0.05 were considered to be statistically significant.

## Results

### IPD serogroup/types

**Figure 2:**
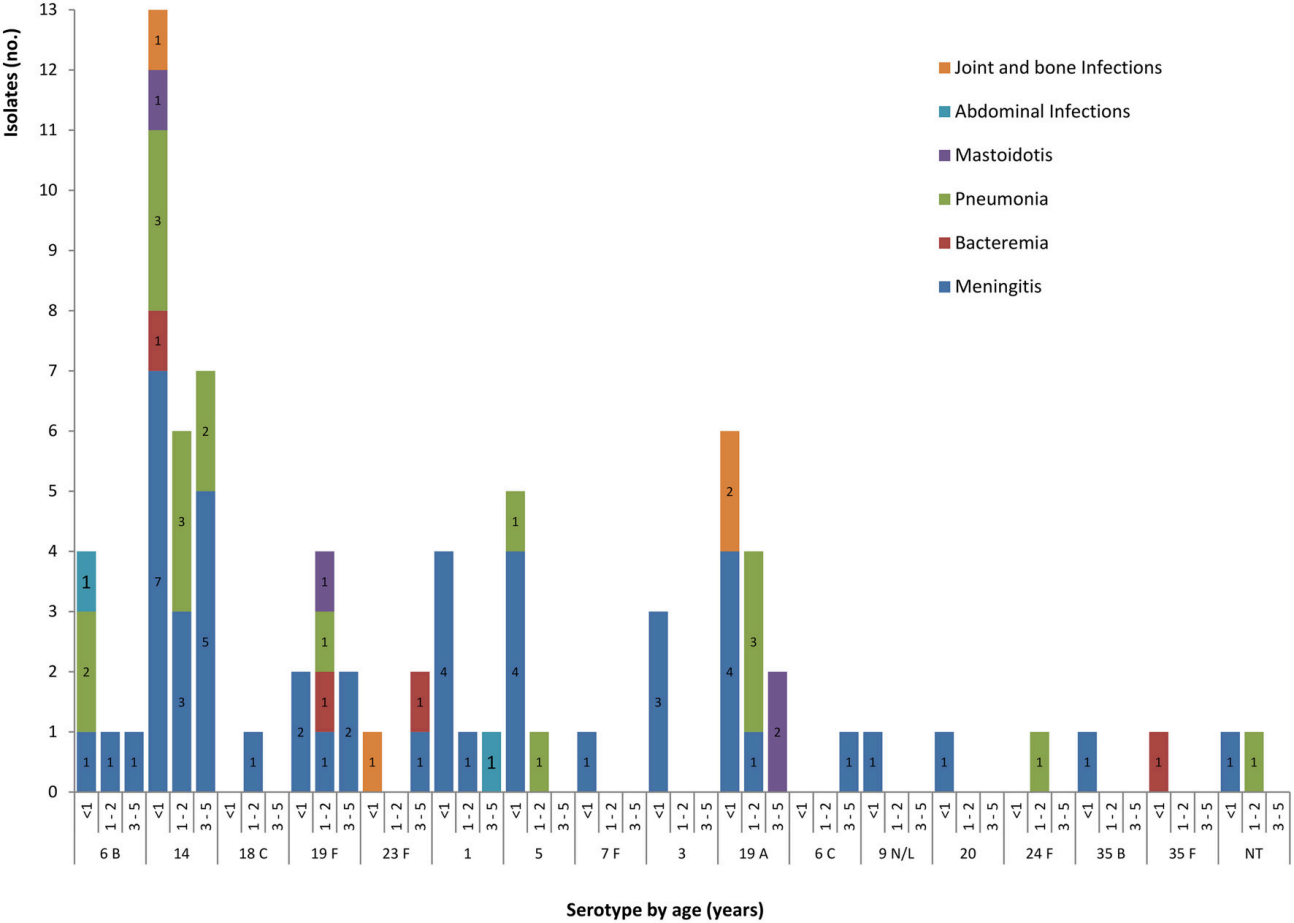
**Serogroup/types distribution and age groups in children under 5 years old according to clinical presentations of invasive pneumococcal disease (IPD) episodes**. Abdominal infections, *S. pneumoniae* was isolated from ascitic and peritoneal fluids; NT, non-typeable.

Between 2010 and 2014, a total of 207 episodes of IPD were registered at LCM/CHU. Among those, 80 (38.6%) corresponded to children <5 years old and were retained for this study. These 80 IPD cases (Figure 2) comprised 56.3% of occurrences in males and 43.7% in females, with 64 cases (80%) being reported to infants (≤2 years old). Overall, the serotypes were identified in 78 isolates, while two were non-typeable (Table 1).

**Table 1:** ***S. pneumoniae* conjugate vaccines PCV7, PCV10, and PCV13 and non conjugate vaccine serotypes implicated in IPD cases and in nasopharyngeal carriage, according to the age groups**.

**Serotypes/Age(years)**	**IPD**				**Nasopharyngeal carriage**				**Global total**	***P*-value^a^**
	**<1**	**1–2**	**3–5**	**Total**	**<1**	**1–2**	**3–5**	**Total**		
**PCV7 SEROTYPES**										
4					1	1	2	4	4	0.161
6 B	4	1	1	6	12	2	2	16	22	0.245
9V/A						1	2	3	3	0.256
14	13	6	7	26	4	4	5	13	39	<0.01
18 C		1		1					1	0.364
19 F	2	4	2	8	6	5	1	12	20	0.449
23 F	1		2	3	8	1	2	11	14	0.182
Subtotal	20	12	12	44	31	14	14	59	103	0.045
**ADDITIONAL PCV10 SEROTYPES**										
1	4	1	1	6	2			2	8	0.028
5	5	1		6					6	<0.01
7 F	1			1					1	0.364
Subtotal	10	2	1	13	2			2	15	<0.01
**ADDITIONAL PCV13 SEROTYPES**										
3	3			3			1	1	4	0.138
6A					11	1	1	13	13	<0.01
19 A	6	4	2	12	5^b^			5^b^	17	<0.01
Subtotal	9	4	2	15	16	1	2	19	34	0.203
**NON CONJUGATE VACCINE SEROTYPES**										
6 C			1	1	1		1	2	3	0.700
8							1	1	1	0.636
9 N/L	1			1					1	0.364
10A					1			1	1	0.636
11A/D					3			3	3	0.256
15							1^c^	1^c^	1	0.636
15 A/F					3	1	2	6	6	0.064
15B/C					5			5	5	0.116
16F					1			1	1	0.636
17F					1			1	1	0.636
20	1			1	1^c^			1^c^	2	0.700
23A					1	1		2	2	0.404
23B					3		1	4	4	0.161
24 F		1		1	1		1	2	3	0.700
33F/A/37					1			1	1	0.636
34					4		1	5	5	0.116
35 B	1			1	3		1	4	5	0.401
35 F	1			1					1	0.364
Subtotal	4	1	1	6	29	2	9	40	46	<0.01
Total Typeable	43	19	16	78	78	17	25	120	198	<0.01
Non typeable	1	1		2	13	3	2	18	20^d^	<0.01
Global Total	44	20	16	80	91	20	27	138	218	

a One-tail P ≤ 0.05 were considered to be statistically significant.

b Four isolates were serotyped by Multiplex PCR and one was serotyped by Quellung reaction.

c Isolates serotyped only by Quellung reaction.

d Three Multiplex PCR non-typeable isolates were serotyped only by Quellung reaction (serotypes 19A^b^, 15^c^, and 20^c^).

**Figure 3:**
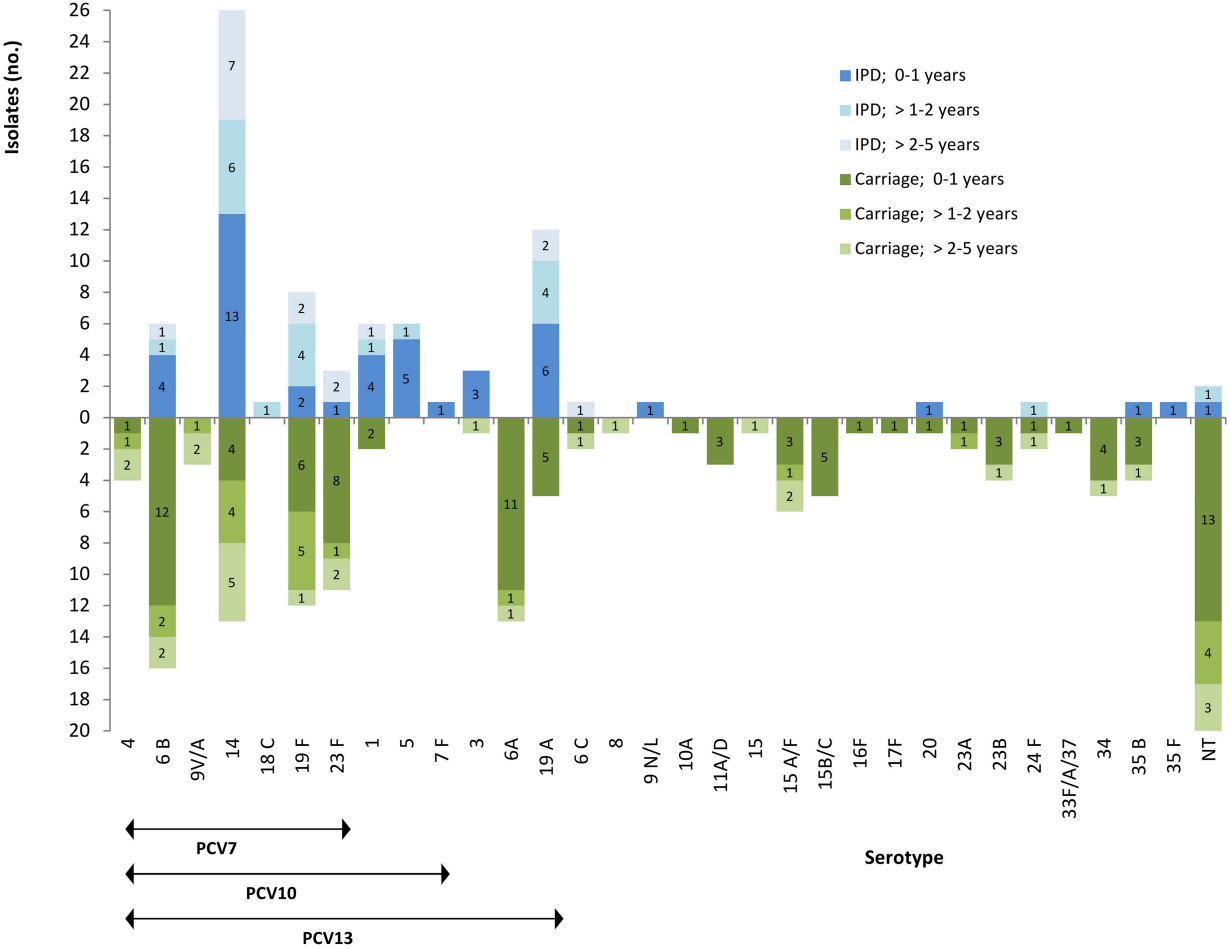
**Distribution of serogroup/types and pneumococcal conjugate vaccine (PCV) coverage for invasive pneumococcal disease (IPD) (*n* = 80) and nasopharyngeal (*n* = 140) isolates in children aged 5 years and less, shown by age group**. NT, non-typeable.

Forty-eight (60%) of the total IPD episodes included cases of meningitis [of which 62.5% (30/48) were in infants <1 year old], while 40% (32/80) comprised cases of nonmeningitis [56.3% of which were pleuropneumonia (18/32), including 55.5% (10/18) in infants from 1 to 2 of age; Figure 2]. The most frequent serotypes implicated in IPD in this study, which accounted for 87.5% (70/80) of the tested isolates, were in decreasing frequency (Figure 3): 14, 19A, 19F, and 1 (from 0 to 5 years old), 5 (from 0 to 2 years old), 6B (from 0 to 5 years old), 3 (from <1 year old), and 23F (from <1 year, and from 3 to 5 years old). Serotypes 14, 1, 19A, 19F, 5, 3, and 6B were the most common in meningitis cases. However, serotypes 14, 19A, 5, and 6B were also implicated in pleuropneumonia, and 19A, 19F, and 14 in bacteremia (Figure 2).

Deaths caused by *S. pneumoniae* infection were noticed in 7 IPD cases (7/80, 8.8%) associated with meningitis (6/7, 85.7%), and pneumonia (1/7, 14.3%). Serotypes 19A and 14 were cause of death in 3 (42.8%), and 2 (28.6%) cases, respectively. The two remaining death cases were caused by serotype 18C, and one non-typeable isolate.

**Figure 4:**
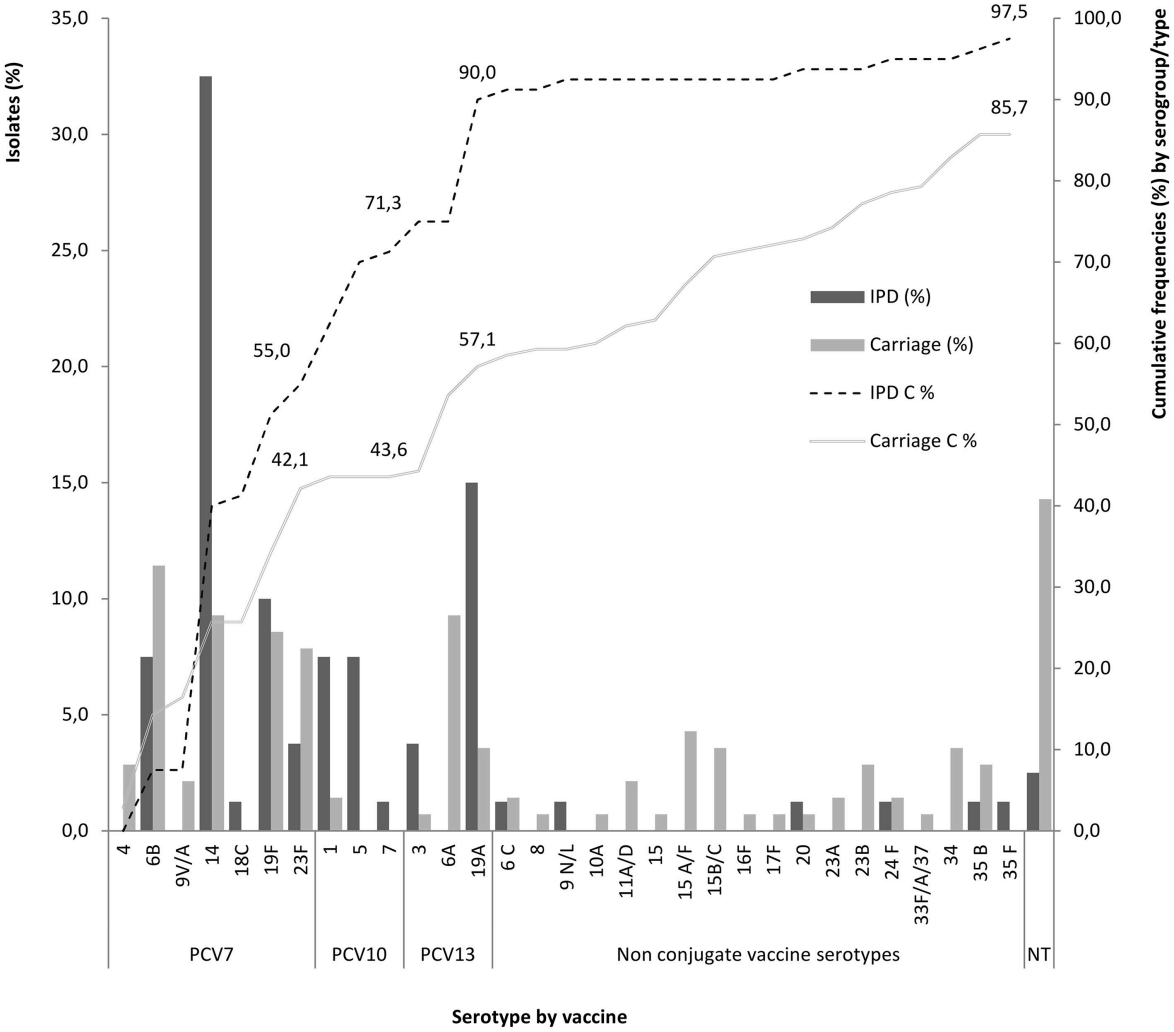
**Rate of serogroup/type cumulative coverage (C %) in each conjugate vaccine (PCV7, PCV10, PCV13) for *S. pneumoniae* recovered from invasive pneumococcal disease (IPD) and asymptomatic carriers less than 5 years old, from Algeria**. NT, non-typeable.

A total of 44 pneumococcal isolates expressed the serotypes included in PCV7, which would reflect the coverage of 55% (44/80) (Figure 4; Table 1). The coverage of PCV10 would be of 71.3% (57/80), because there were 13 isolates expressing serotypes 1 (6/57, 10.5%), 5 (6/57, 10.5%), and 7F (1/57, 1.8%). The coverage of PCV13 would reach up to 90% (72/80) (Figure 4, Table 1). All PCVs would cover the three age groups included in the study. Non-vaccine serogroup/types were expressed in 7.5% (6/80) with one isolate of each: 6C/D, 9N/L, 20, 24F, 35B, and 35F.

### Serogroup/types characterization of nasopharyngeal isolates

The serogroup/type of 138 isolates, identified in 130 nasopharyngeal cultures from colonized children <5 years old, was determined by multiplex PCR method, using the same scheme as for IPD isolates (Figure 1): 117 (83.6%) *S. pneumoniae* were characterized at the serogroup/type level [101 (86.3%) children carried a single serogroup/type, whereas eight children (8/132, 6.1%) were co-colonized with pneumococci belonging to two different serogroup/types]. Furthermore, of the 18 isolates that remained non-typeable by multiplex PCR reactions, 3 were assigned to serogroup/type 15, 19A, and 20 by the Quellung reaction (Table 1). Serogroup 6A/B was differentiated by Quellung reaction into serotype 6B (*n* = 16) and serotype 6A (*n* = 13).

The most common serogroup/type was 6 (*n* = 31) [divided in 6A (*n* = 13), 6B (*n* = 16) (both included in PCV13 and PCV7, respectively), and 6C/D (*n* = 2)]; it was followed by serogroup/type 14 (*n* = 13), 19F (*n* = 12), and 23F (*n* = 11; Figure 4; Table 1). All these four serogroup/types were identified in the three age groups defined: <1 year, 1–2 years, and 3–5 years (Figure 3). The remaining conjugate vaccine serotypes identified in asymptomatic children carriers of *S. pneumoniae* included 19A (*n* = 5), 4 (*n* = 4), 9V/A (*n* = 3), 1 (*n* = 2), and 3 (*n* = 1). Non-conjugate vaccine serotypes accounted for 29% (40/138), identified by both PCR (*n* = 38) and Quellung reaction (*n* = 2). These serotypes were mostly identified as 15A/F, 15B/C, 34, 23B, 35B, and 11A/D. Globally, the section of the study that included the carrier patients allowed the identification of 120 isolates at serogroup/type level, and the evaluation of 18 non-typeable isolates.

Overall, 4.1% (09/218) of the isolates lacked the *cpsA* gene. However, the presence of the *lytA* gene confirmed the identification of *S. pneumoniae* specie. In addition, the sequential multiplex PCR scheme (Figure 1) used in our study demonstrated a specific identification of each serogroup/type in 90.8% (198/218) of the isolates analyzed: among the 80 IPD and the 138 nasopharyngeal *S. pneumoniae* isolates, 97.5% (78/80) and 86.9% (120/138) were positively serotyped by sequential multiplex PCR, respectively (*p* < 0.01; Table 1).

### Antibiotic susceptibility and vaccine serotype

Among *S. pneumoniae* vaccine serotypes (*n* = 72) from meningitis (*n* = 39) we identified 66.7% isolates penicillin non-susceptible (MIC ≥ 0.12 μg/ml) with 25.6% showing high level of resistance (MIC 2–4 μg/ml; Table 2). Isolates non-susceptible to cefotaxime and imipenem corresponded to 40.5 and 43.2%, respectively. However, high level of resistance was noticed for imipenem only in 5.4% isolates.

**Table 2:** **Antibiotic susceptibility of 72 isolates of vaccine serotypes^a^, according to site infection**.

**Antibiotic^b^^,^^c^ site of infection**	**Antibiotic susceptibility (%)**				**Total no. of isolates (*n* = 72)**	**Breakpoint**^b^^,^^c^ **(CLSI**, **2014****)**	
	**S**	**I**	**R**	**IR**		**S≤**	**R≥**
**PENICILLIN G**							
Meningitis	33.3	–	66.7	66.7	39	0.06	0.12
**PARENTERAL PENICILLIN PG**							
No meningitis	88.0	8.0	4.0	12.0	25	2	8
**ORAL PENICILLIN**							
No meningitis	20.0	32.0	48.0	80.0	25	0.06	2
**AMOXICILLIN**							
No meningitis	86.4	9.1	4.5	13.6	22	2	8
**CEFOTAXIME**							
Meningitis	59.5	40.5	0.0	40.5	37	0.5	2
No meningitis	91.7	8.3	0.0	8.3	24	1	4
**IMIPENEM**							
Meningitis	56.8	37.8	5.4	43.2	37	0.12	1
**ERYTHROMYCIN**							
Meningitis	57.5	0.0	42.5	42.5	40	≥21	≤15
No meningitis	25.9	0.0	74.1	74.1	27	≥21	≤15
**CLINDAMYCIN**							
Meningitis	60.0	0.0	40.0	40.0	40	≥19	≤15
No meningitis	33.3	0.0	66.7	66.7	27	≥19	≤15
**CO-TRIMOXAZOLE**							
Meningitis	46.2	2.6	51.2	53.8	39	≥19	≤15
No meningitis	25.0	0.0	75.0	75.0	24	≥19	≤15
**TETRACYCLINE**							
Meningitis	71.8	2.6	25.6	27.2	39	≥28	≤24
No meningitis	57.7	3.8	42.3	46.2	26	≥28	≤24

a Includes serogroups/types: 1 (n = 6), 3 (n = 3), 5 (n = 6), 6B (n = 6), 7 (n = 1), 14 (n = 26), 18C (n = 1), 19A (n = 12), 19F (n = 8), and 23F (n = 3).

b Susceptibility testing by E-test method (MIC determination), for penicillin, amoxicillin, cefotaxime, and imipenem.

c Susceptibility testing by agar disk diffusion method, for erythromycin, clindamycin, co-trimoxazole, tetracycline.

S, Susceptible; I, Intermediate; R, resistant; IR, non-susceptible.

From no meningitis infections (*n* = 25), 80% of the isolates were penicillin non-susceptible with 12% of high level of resistance (MIC = 4–8 μg/ml). Intermediate resistance to amoxicillin and cefotaxime was 9.1 and 8.3%, respectively. High level of resistance to amoxicillin was observed in 4.5% isolates (MIC 8 μg/ml).

Non-susceptibility to erythromycin, clindamycin, co-trimoxazole, and tetracycline occurred in lower frequencies in isolates from meningitis (42.5, 40, 53.8, and 27.2%, respectively) than in isolates from no meningitis infections (74.1, 66.7, 75, and 46.2%, respectively).

High level of resistance for vaccine serotypes was observed in serotypes 14, 19A, 19F, and 23F. Overall, the vaccine serotypes 14, 19A, 19F, 23F, 6B, and 5 presented higher rates of resistance (90–100%) than the eight non-vaccine serotypes (6C, 9N, 20, 24F, 35B, 35F, non-typable; *p* < 0.05; data not shown).

## Discussion

*S. pneumoniae* remains the leading cause of bacterial infection among children worldwide, including numerous cases of invasive disease associated high morbidity and mortality rates (Harboe et al., 2010; Adegbola et al., 2014). The pneumococcal epidemiology regarding capsular types and antibiotic resistance varies geographically and temporally in terms of origin of the isolates (infections or carriage), clinical presentation, pathogenicity (Hausdorff et al., 2005; Harboe et al., 2010; Ingels et al., 2012; Geno et al., 2015), and the methods used for serotyping (Turner et al., 2011; Song et al., 2012; Geno et al., 2015). Although antibiotic susceptibility and serotype data remains insufficient in many countries such as Algeria, its collection and evaluation is essential for the treatment of pneumococcal infections and for the usage of conjugate vaccines (Ramdani-Bouguessa and Rahal, 2003; Tali-Maamar et al., 2012; Ziane et al., 2012; Ramdani-Bouguessa et al., 2015).

Thus, the main goal of this study was to analyze the frequency of serotypes associated to IPD and *S. pneumoniae* asymptomatic carriage in Algeria, in children <5 years old before the introduction of the pneumococcal vaccine. To accomplish our purpose, we used the more recent IPD samples studied in the country (Ramdani-Bouguessa and Rahal, 2003; Tali-Maamar et al., 2012; Ziane et al., 2012; Ramdani-Bouguessa et al., 2015). It is worth mentioning that a molecular approach was here applied for the first time to isolates recovered in this country in the context of a monitoring survey of *S. pneumoniae* serogroup/types (Ziane et al., 2015).

In this study, meningitis represented 60% of the clinical presentation of IPD, with 79.2% registered in children younger than 2 years old. Pleuropneumonia was reported in 22.5% of the total IPD cases, with the majority being manifested in infants (88.8%, 16/18). Differently from bacteremia, mastoiditis, and abdominal infections, the bone and joint infections were noted exclusively in infants with up to 1 year old. Indeed, IPD occurs mostly in children under the age of 5 years, especially within the subgroup of those under 2 years of age (Tan, 2012). The prevalence of *S. pneumoniae* carriage in healthy children younger than 5 years old can fluctuate between 20 and 93.4% in low income countries, being superior than what is stated for lower-middle income countries (from 6.5 to 69.8%), as reported by Adegbola et al. (2014). In addition, the density of pneumococcal nasopharyngeal carriage seems to decrease in higher age groups, with the children being more competent than adults in the transmission of pneumococci (Roca et al., 2012).

Mortality rate of pneumococcal invasive diseases may range from 10 to 30%, according with the studies of Pebody et al. (2006), and Bravo (2009). Although it has been reported that the highest *S. pneumoniae*-associated morbidity and mortality rates are in Africa and Asia (Johnson et al., 2010; Turner et al., 2011), in our study the case fatality was 8.8%, mainly comprising meningitis related with vaccine serotypes (85.7%).

Overall, we identified serogroups 14, 19, 23, and 6 as the most common among IPD and nasopharyngeal carriers (Figure 2). Among IPD isolates, serotypes 14, 19A, 19F, 6B, 1, and 5 were the most frequent, which shows some differences from data described for other periods in Algeria. For instance, the serogroups/types 1 and 5 were the most common for the period of 1996–2000 (Ramdani-Bouguessa and Rahal, 2003), the serogroups/types 14, 19F, 23F, and 6B for the periods of 2001–2010 and 2005–2011 (Tali-Maamar et al., 2012; Hecini-Hannachi et al., 2014), and serogroups/types 14, 19F, 6B, 1, and 19A for the period of 2005–2012 (Ramdani-Bouguessa et al., 2015). In Tunisia, the most prevalent serogroups for IPD in children were nearly the same: 19, 14, 23, and 4 (Charfi et al., 2012). Internationally, among the 98 known pneumococcal serotypes 11 of them account for more than 70% of IPD, in children younger than 5 years old, with serotypes 1, 5, 6A, 6B, 14, 19F, 23F being the most common (Johnson et al., 2010). Indeed, it has been described that a restricted number of serotypes is in the origin of the majority of the IPD cases worldwide. To summarize, since the report of the first studies concerning the distribution of IPD serotypes in Algeria, there was an emergence of serotype 19A, which is one of most common causes of invasive disease in developed countries in children (Geno et al., 2015). Furthermore, the studied isolates expressing serotype 19A were assigned to ST276 (M. Caniça, personal communication), which is one of the most predominant sequence-types within this serotype (Reinert et al., 2010; Ramos et al., 2014).

Some of the *S. pneumoniae* serotypes are more prone to successfully colonize the nasopharynx, being in advantage to cause invasive disease. The carriage of *S. pneumoniae* may play an important role in the pathogenesis of IPD and in the transmission of this bacterium (Roca et al., 2012). Indeed, vaccination often brings a decrease in the reduction of vaccine serotype *S. pneumoniae* isolates, and a raise in carriage of non-vaccine serotype isolates (Dias and Caniça, 2007).

In this study, it is essential not only to emphasize the differences in serotype distribution (in IPD and asymptomatic carriage), but also to consider the presence of non-vaccine types (such as 15, 35B, and 34), particularly in carriage, despite the unavailability of pneumococcal vaccines in Algeria (Figure 2). Thus, when comparing serotype rate for IPD and nasopharyngeal carriage, serotypes 14, 1, 5, and 19A were more implicated in IPD than in carriage (*p* < 0.01), whereas serotype 6A was exclusively isolated from carriers (*p* < 0.01; Table 2).

Non-typeable isolates by PCR methods should be typed by Quellung reaction in order to monitor and detect the emergence of *S. pneumoniae* serotype variants, and if this test is negative, the isolates should be sequenced for the *cps* locus to characterize variants (Bentley et al., 2006). In fact, the *cpsA* gene is common within most encapsulated *S. pneumoniae*, being a highly conserved region of the *cps* locus in all known pneumococcal *cps* operons (Bentley et al., 2006; Pai et al., 2006; Jin et al., 2009). However, the absence of *cpsA* amplification in the multiplex PCR scheme has been reported among rough strains, and pneumococci with mutated capsular genes, or without the *cps* locus (Pai et al., 2006; Ahn et al., 2012; Richter et al., 2013). Previous studies have also reported absence of *cpsA* gene by PCR-based serotyping of *S. pneumoniae* in 1–3% of the cases, particularly among serotypes 38 and 25F (da Gloria Carvalho et al., 2010; Jourdain et al., 2011). In this study, 4.1% of the isolates lacked the *cpsA* gene.

Among the *cpsA* negative isolates, the detection of the *lytA* gene resolved the *S. pneumoniae* specie (Moreno et al., 2005). Thus, we propose the scheme in Figure 1 for pneumococcal serotyping that will extend the application of multiplex PCR in laboratories, and concentrate the use of conventional method in reference laboratories only.

Similarly to other countries (Flasche et al., 2011; Ahn et al., 2012; Steens et al., 2013; von Gottberg et al., 2014), we noticed in Algeria an increase of serotypes covered by the different PCVs, particularly among IPD (Figure 4). These new data suggests an increase in the frequency of serotypes included in PCV13 in Algeria, in comparison with the 74.2% registered between 2005 and 2011 (Hecini-Hannachi et al., 2014), but similar to what was observed from 2005 to June 2012 (86.8%; Ramdani-Bouguessa et al., 2015).

Concerning the antibiotic resistance of *S. pneumoniae* from vaccine serotypes, the rates obtained were higher in no meningitis infections probably due to the large use of these antibiotics in the treatment of respiratory or urinary tract infections in Algeria. Tetracycline resistance rate might be explained by a lesser use of this antibiotic.

Rates of *S. pneumoniae* non-susceptible to penicillin vary in the country according to the study periods. Therefore, Algerian studies reported 34.6% of penicillin non-susceptible *S. pneumoniae* in 2003 (Ramdani-Bouguessa and Rahal, 2003), 25.2% in 2001–2010, mostly from meningitis, and 4.4% of high level resistance to cefotaxime (Tali-Maamar et al., 2012). A later study, conducted on invasive and non-invasive pneumococcal disease in children, identified 48% of penicillin non-susceptible isolates, mainly in no meningeal infections (Ramdani-Bouguessa et al., 2015). Rates of 48.5% and 45% have been reported in Morocco and Tunisia, respectively (Charfi et al., 2012; Elmdaghri et al., 2012).

In our study, the decreased susceptibility to penicillin is significantly associated with both vaccine serotypes and non-vaccine serotypes (*p* < 0.05; data not shown), but the vaccine serotypes 14, 19A, 19F, and 23F, showed the highest levels of penicillin resistance (MIC Peni G > 2μg/ml). These data is in agreement to earlier reports from Algeria (Ramdani-Bouguessa and Rahal, 2003; Hecini-Hannachi et al., 2014), Tunisia (Charfi et al., 2012), Morocco, (Elmdaghri et al., 2010), and France (Varon, 2012), where the serotypes 14, 19F, 23F, 6B were described as the most resistant. However, in France, after the introduction of PCV13, the serotypes 19A, 19F, 15A, 35B, and 24F emerged as those with higher resistance (Varon et al., 2015).

The fluctuations of *S. pneumoniae* circulating serotypes and its relation with antibiotic resistance and the PCVs coverage, reinforces the importance of *S. pneumoniae* serogroup/type identification and studies of antibiotic susceptibility, in pre- and post-vaccination periods, particularly in countries with few data, such as Algeria. These will help guide the treatment and will motivate the implementation of strategies for prevention of pneumococcal disease.

## Author contributions

HZ designed the study, performed experiments, analyzed the data, and wrote the manuscript. VM analyzed the data and reviewed the manuscript. EF performed experiments and reviewed the manuscript. IM performed experiments and reviewed the manuscript. SB performed experiments. MT reviewed the manuscript. MC designed the study, analyzed the data and reviewed the manuscript. All authors read and approved the final manuscript.

## Funding

HZ was supported by a grant from Ministère de l'Enseignement Superieur et de la Recherche Scientifique in Algeria. VM was supported by FCT fellowship (grant SFRH/BPD/77486/2011), financed by the European Social Funds (COMPETE-FEDER), and national funds of the Portuguese Ministry of Education and Science (POPH-QREN).

### Conflict of interest statement

The authors declare that the research was conducted in the absence of any commercial or financial relationships that could vbe construed as a potential conflict of interest.
